# JNets: Exploring networks by integrating annotation

**DOI:** 10.1186/1471-2105-10-95

**Published:** 2009-03-26

**Authors:** Jamie I MacPherson, John W Pinney, David L Robertson

**Affiliations:** 1 Faculty of Life Sciences, Michael Smith Building, University of Manchester, Oxford Road, Manchester, M13 9PT, UK; 2 Division of Molecular Biosciences, Imperial College London, London, SW7 2AZ, UK

## Abstract

**Background:**

A common method for presenting and studying biological interaction networks is visualization. Software tools can enhance our ability to explore network visualizations and improve our understanding of biological systems, particularly when these tools offer analysis capabilities. However, most published network visualizations are static representations that do not support user interaction.

**Results:**

JNets was designed as a network visualization tool that incorporates annotation to explore the underlying features of interaction networks. The software is available as an application and a configurable applet that can provide a flexible and dynamic online interface to many types of network data. As a case study, we use JNets to investigate approved drug targets present within the HIV-1 Human protein interaction network. Our software highlights the intricate influence that HIV-1 has on the host immune response.

**Conclusion:**

JNets is a software tool that allows interaction networks to be visualized and studied remotely, from within a standard web page. Therefore, using this free software, network data can be presented in an enhanced, interactive format. More information about JNets is available at .

## Background

Interaction networks can be studied to gain a greater understanding of the biological system that they represent [1-4]. A common method for studying PPI networks is through visualization, typically by representing a network as a 'ball-and-stick' graph. Interactive visualizations can enhance our understanding of networks and allow new patterns and trends to be discerned [5], particularly when these tools offer network analysis capabilities. However, most published PPI network visualizations are static representations that do not permit the user to view associated annotation let alone integrate and analyze other biological information in a useful manner.

The development of JNets was motivated by the need for an online, interactive protein-protein interaction (PPI) network viewer for the HIV-1, Human Protein Interaction Database (HHPID) [6,7]. HHPID is a valuable resource for the study of HIV-1 infection. HHPID data is manually curated and in addition to the pairs of interacting HIV and human genes, contains details of the interaction type (e.g., 'phosphorylates' or 'complexes with'). Currently, many network visualization software tools are available that facilitate both interaction and analysis for example [8-13]. However, a flexible tool is required that can be deployed from a website as an applet, that combines network visualization and manipulation capabilities with analysis methods focused on biological annotation (such as the HHPID interaction type). This tool will aid in understanding the mechanism of infection and the host-viral interactions involved in the HIV-1 life cycle but will also be useful for a wide range of network-related projects, biological or otherwise.

JNets is available as a stand-alone application and a web-deployable applet and is applicable to any type of biological or non-biological network data. Analysis in JNets is achieved by overlaying node and edge annotation on to the network. Groups of nodes and edges can be created by filtering accompanying annotation, and properties of groups can be explored, in terms of annotation, both visually and statistically. In addition, JNets is configureable to allow web-deployed visualizations to be customized by a vendor. Specifically, preset network visualizations can be defined and the JNets user interface altered. Furthermore, JNets is free and platform independent.

## Implementation

**Figure 1:**
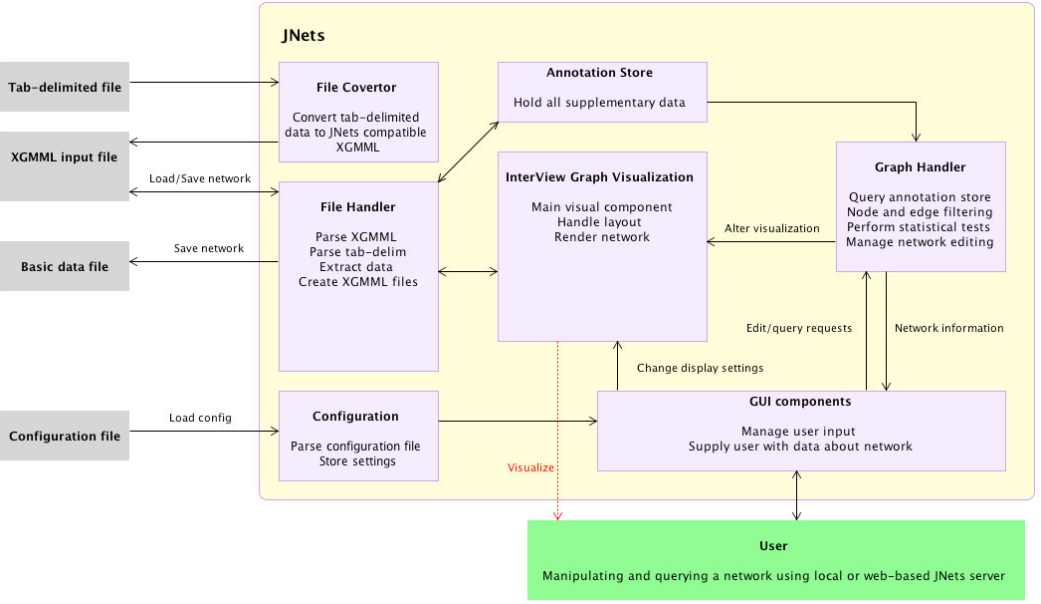
**Diagrammatic representation of the the JNets system**. This diagram describes the conceptual flow of information through the software.

JNets is available in two forms: a stand-alone application and a web-deployable applet. The latter has some features disabled (such as the 'File menu') due to the security requirements of Java applets. Certain advantageous features in JNets were inherited from InterView [12], the software on which JNets is based. These include the animated spring layout, a container layout [12], interactive 'clickable' nodes and the facility to export network visualizations in PDF and PNG formats. In addition, the Java libraries responsible for graph layout, network rendering and the legend panel also come from InterView. InterView uses libraries from the TouchGraph package. These drive the interactive network display in JNets. A diagrammatic summary that shows the organization of JNets is given in figure 1. JNets is available from , where an applet can be launched to visualize and browse the HHPID network. Also available at this site is a download package, including source code, documentation and example data files.

## Results

**Figure 2:**
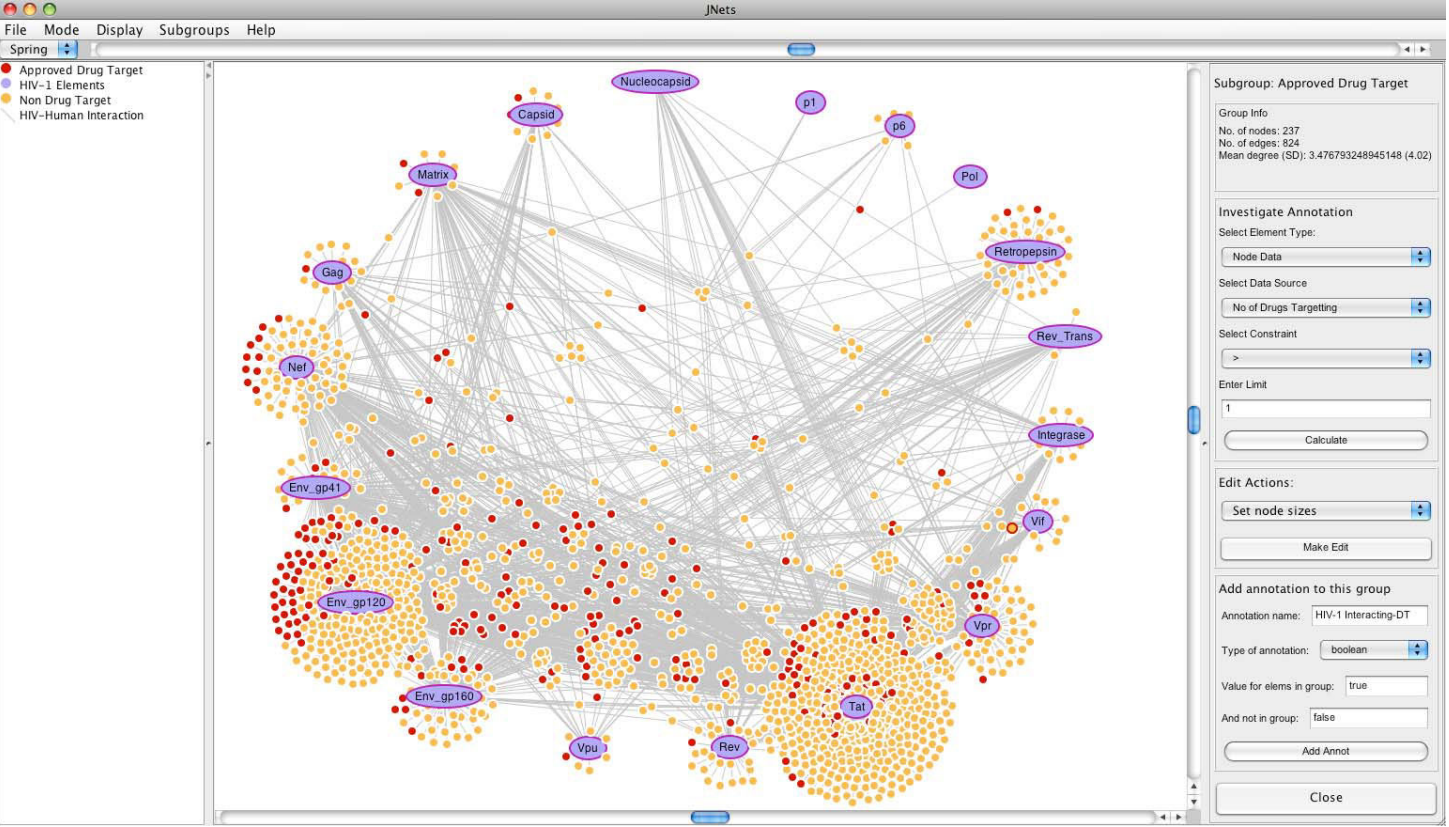
**The JNets user interface**. The main interface and network visualization panel from the JNets application is shown. JNets is displaying the HIV-1 human PPI network, from HHPID [6,7]. On the left is the legend panel showing the node and edge groups. The menu bar at the top is customizable. Standard drop-down menus can be enabled or disabled and new menus with user-defined network views can be added, defined in the configuration file. On the right the subgroup edit and analysis panel is shown, through which the user can tailor the visualization, explore subgroup annotation and add further annotation to the network.

The following sub-sections describe the main features of JNets in detail. Where appropriate, examples are given using network data from HHPID [6,7]. The main JNets interface is described in figure 2.

### Input files

JNets requires a main input file in Extensible Graph Markup and Modeling Language (XGMML) format. This XML format was chosen because it is designed to record network structures and includes support for node and edge annotation. To aid the creation of JNets-compatible XGMML files, a tool for converting tab-delimited data is provided in the JNets application. These input XGMML files hold three types of information: (1) data that are essential to the network structure, for example, node and edge identifiers, node and edge names, edge directionality and edge source-node and target-node identifiers. These data are sent directly to components from InterView to create and visualize the network. (2) Optional attributes of the network display, such as x and y coordinates for nodes to enable predefined layouts, initial node and edge groupings and whether node labels are displayed on startup. (3) Annotation, i.e., any additional data attributed to a node or edge.

### Element Annotation

In JNets, nodes and edges can be given any number of different annotations, these annotations can be used for network analysis. Many other network visualization tools offer annotation of graph elements, however, these are often linked to specific, predetermined biological resources. For example VisAnt integrates information from the GO and KEGG databases [14]. For maximum flexibility and ease of use, annotation in JNets is entirely determined by the user. These annotations fall in to two classes, either *single *or *multiple*. Single annotations are those where only one value can be attributed to an element. For example, nodes, representing proteins in HHPID, could be annotated with a source organism taxonomic identifier. Only one such value will be attributed to each protein, either human or HIV-1. Therefore, taxonomic identifier could be defined using a *single *annotation. *Multiple *annotations are those where a single element may have multiple values for one annotation. For example, in HHPID every PPI is attributed with a list of PubMed article identifiers that report the interaction. As this can be one or multiple articles, PubMed IDs would be defined using a *multiple *annotation. Annotations can also take different types of value; supported types are strings, integers, decimal values (doubles) or boolean values (true or false). Annotations are handled according to their class and value type. This overall system of annotation allows nodes and edges in JNets to be linked to a wide range of data, biological or otherwise. Right clicking on a node or edge allows an element information panel to be viewed. These information panels display all the annotation that has been loaded for any element, along with some basic data such as the number of neighbors a node has, the element identifier and label values.

### Subgroup Creation

**Figure 3:**
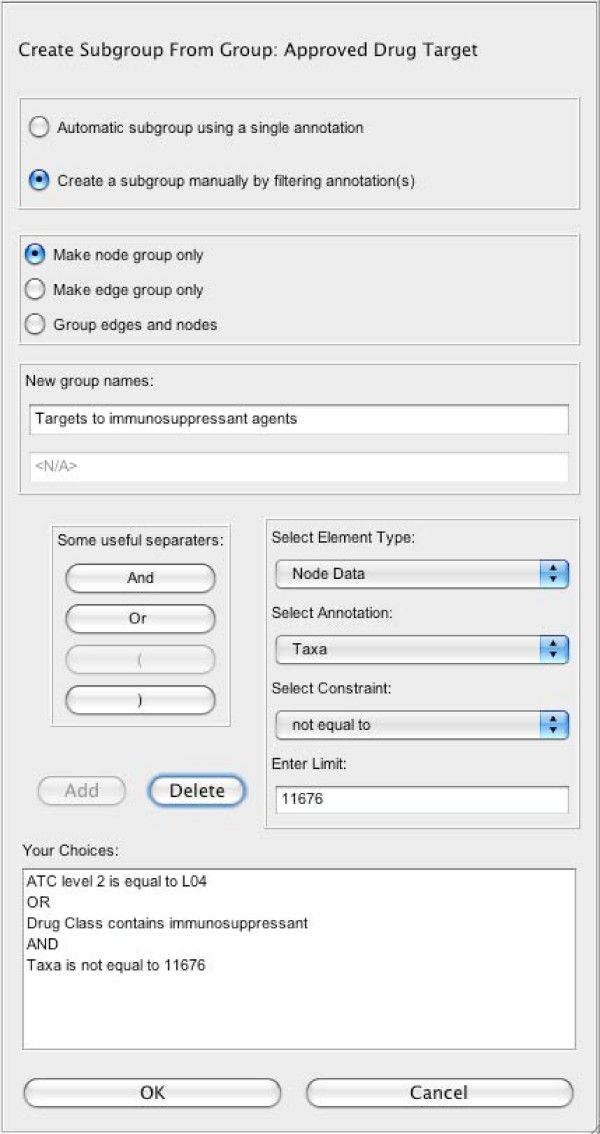
**The JNets subgroup creation interface**. This interface is used to create a new subgroups from already existing ones. The upper half of this panel is used to select some simple options about the subgroups being created, such as whether the grouping should be made automatically or manually, what the name of new subgroups should be and whether new a new node group, edge group, or both should be created. The lower half of this panel is used to select the annotation and set the filters through which new subgroups will be created.

An integral feature of JNets is the ability to edit and investigate subgroups of elements. In the other network viewers, JSquid [13] and InterView [12], subgroups are possible but are not dynamic, as they are predetermined in the input file. However, in JNets, users can create novel subgroups of elements using a simple, flexible system of filtering element annotation. To create a subgroup, three main components are considered. Firstly the input group. This can be the whole network or a previously created edge group, or node group. Note, the input group is the set of elements that will be filtered. Secondly, the element filter. There are two types of filter in JNets: *automatic *filters and *manual *filters. *Automatic *filters create an array of subgroups by taking the input group and dividing elements according to the value that they hold for a *single *annotation. For example, in HHPID, proteins could be automatically filtered according to their taxonomic identifier. This would produce two subgroups of proteins: human and HIV-1. *Manual *filters operate like SQL select statements, where the input group is filtered according to any number of constraints concatenated with AND and OR operators, the output being elements that are true for that statement. For example, the interactions in HHPID could be filtered to find those that are annotated as binding OR activation but NOT phosphorylation interactions. JNets also allows nodes to be filtered according to edge annotation (and vise versa) by taking in to account edge incidences at each node. This greatly increases JNets flexibility in creating subgroups. For example, by this method, human *proteins *could be filtered according to whether they take part in an *interaction *that is annotated as binding OR activation but NOT phosphorylation. The third component is the output type. This can be nodes, edges or both nodes and edges. The output type determines the type of element that is subject to regrouping. For example, HIV-1 proteins could be filtered to find the HIV-1 accessory proteins Vpr and Vpu. Given an output type of nodes *and *edges this would result in the creation of a new node group consisting of Vpr and Vpu and a new edge group of interactions involving Vpr and Vpu. Therefore, JNets allows users great freedom to create subgroups of interest. By creating and visualizing network subgroups, patterns in the data (that link network annotation and network structure) may more easily be identified. Pattern identification may lead to generation of hypotheses concerning the distribution of annotation values over the network. These hypotheses can be tested using the network analysis methods available in JNets. The JNets subgroup creation interface is shown in figure 3.

### Network Analysis

JNets provides simple but powerful methods for analyzing networks and the annotations that are attributed to network elements. The main analytical capability in JNets is to examine subgroups of elements and assess annotation content using statistical methods. For example, using only the HHPID data and JNets, it is possible to create a subgroup of HIV-1-human interactions that involve the Tat protein and discover that there are 774 such interactions from a total of 2,588 unique PPIs.

Furthermore, the most statistically significant over-represented interaction type in this group is 'enhances', with a total of 53 interactions (*p *= 3.3 × 10^-15^), using all interactions as the general population, including a correction for multiple tests. To produce such results, JNets will either perform a two-tailed Fisher's Exact test, or Chi-Square approximation test, depending on the population size. The hypergeometric and chi-squared distributions required to do these tests are calculated using classes from the open source Colt 1.2.0 Java package. Where appropriate, correction for multiple significance testing is implemented automatically, using the Benjamini-Hochberg false discovery rate method [15].

This system of analysis allows JNets to remain both flexible (as both the annotation and the subgrouping used in the analysis are determined entirely by the user) and fast (as these calculations are computationally simple), particularly important for deployment as an applet. Furthermore, as calculations are performed directly upon visible subgroups of the network, a link between the visual and statistical output of JNets is explicit. The following case study demonstrates the power of these analysis methods.

### Network Manipulation

A key feature of JNets is the ability to manipulate the network to improve visualizations. Basic but very useful manipulations available in JNets include setting node and edge colors, setting node sizes, turning node labels on or off, collapsing parts of the network to form composite nodes, deleting elements and sticking nodes to prevent the spring layout from acting upon them. Most of these features were previously available in InterView, but acted upon the entire network only. With JNets, these actions can also be applied to novel subgroups of nodes or edges, created by filtering network annotation, to tailor the visualization to the requirements of the user.

### Customizing JNets

JNets can also be customized by providing a configuration file. The configuration file is also in XML format. There are three main aspects of JNets configuration. Firstly, simple user-interface customization. Much of the user interface can be disabled including drop-down menus and right-click functions and the legend panel can be hidden or shown. The ability to create novel subgroups of elements can be disabled, as can specific parts of the subgroup edit and analysis panel. Secondly, the configuration file can determine what data JNets will use from the input XGMML file, in terms of nodes, edges and annotations. This configuration feature allows a single input file to be used, with a number of configurations, to produce a number of different networks. Thirdly, and most importantly, preset network views can be determined in the configuration file. Network views are defined in the configuration file as sets of annotation filters that encode the creation of new node and edge subgroups in the network. These presets allow groups of nodes and edges to be hidden, so that specific parts of the network can be highlighted. The presets appear in new drop down menus on the main menu bar and on a mouse click will execute the filters to alter the network view.

### Case study: Drug targets in the HIV-host network

To demonstrate the utility of JNets as a network visualization and analysis tool, human genes that code for products that are both HIV-1 interacting and approved drug targets were examined, using HHPID as a source for HIV-1-host interactions.

#### Case study: Introduction

To date, only one U.S. Food and Drug Administration (FDA) approved therapeutic agent, maraviroc, developed by Pfizer, directly targets a host, rather than a viral protein in the treatment of HIV-1 infection. Maraviroc is an antagonist of the CCR5 chemokine receptor and prevents CCR5-tropic strains of HIV-1 from binding this co-receptor and entering host immune cells [16]. Other HIV-1 therapeutic agents may follow the lead of maraviroc and target host, rather than viral proteins, in the treatment of HIV-1 infection. For example, HMG-CoA reductase inhibitors (statins) are normally used to treat high cholesterol but have been shown to increase CD4+ cell counts and decrease viral load in HIV-1 infected patients. This action is thought to be due to the negative regulation of Rho GTPase activity by statins, impeding viral entry and budding from the cell [17], further evidence that targeting host proteins can disrupt HIV's biology. It is also possible to target HIV-host interactions directly. For example, disrupting the Vif-APOBEC interaction with a small-molecule, RN-18, is a strategy that is currently being pursued [18] because Vif mediates the degradation of the potent anti-retroviral APOBEC proteins (see review [19]) and in the absence of Vif, HIV-1 virions are non-infective [20].

In this case study we use JNets to explore the HIV-1-interacting proteins that are approved drug targets, as this may provide a useful insight for researchers investigating new therapeutic strategies to treat HIV-1 infection and, at the very least, indicate that a human protein that HIV interacts with is to some extent "druggable". Moreover, close examination of the drug classes and drug targets in this intersect may highlight ways that HIV-1 acts to perturb the host system. We consider both the details of the HIV-1 interactions and the types of drugs that target these human proteins.

#### Case study: Methods

HHPID data were downloaded from the National Centre for Biotechnology Information (NCBI) on May 1st, 2008. These data included the update to HHPID that includes Env gene-product interactions (available November 13th, 2007). The HIV-1 interaction type was taken from the 'interaction' column of this data file. FDA approved drug and drug-target information was taken from the downloadable file 'drugcard set.txt' from the DrugBank database [21,22] on June 3rd, 2008. Drug categories were extracted from the 'Drug_Category' field and the Anatomical Therapeutic Chemical (ATC) classification system code was taken from the 'ATC_Codes' field of the same file.

A single HIV-1-host, drug-target network was created to conduct the investigation. The nodes in this network consisted of all FDA-approved drugs and all FDA-approved drug-target genes, all human genes that code for an HIV-interacting product and all HIV-1 elements (the term 'elements' is used because some of these are genes and proteins). Genes present both in the drug-target and HIV-1 interacting groups were only represented by a single node. There were two types of edges in this network: HIV-host interactions and drug-target interactions. HIV-host interactions came from HHPID. There were 3,939 distinct interactions between the nodes of HIV-1 and the host, distinct on the HIV-1 element, the host gene and the interaction type. Therefore by this definition, interactions that are reported in multiple source articles only count as one interaction. However, multiple interactions between the same nodes are possible as these may have significantly different interaction types, e.g., 'upregulates' and 'downregulates'. The nodes in the network were annotated, where relevant, with drug classifications and ATC codes, the number of distinct HIV-1 interactors and the number of HIV-1 interactions. The edges in the network were also annotated where relevant, with information derived from HHPID, such as the interaction type and whether the interaction is agonistic, antagonistic or neither.

All results were produced by visualizing and analyzing the HIV-1-host, drug-target network using the JNets application.

#### Case study: Results

**Figure 4:**
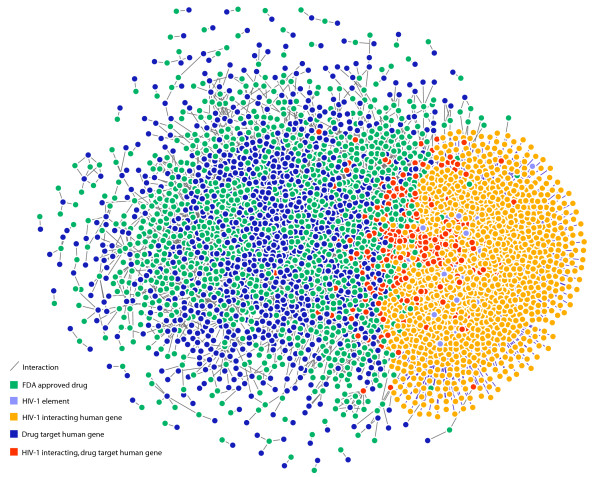
**The HIV-1-host, drug-target interaction network**. This is the whole network that was used for all analyses in our case study. Using the annotation that accompanies nodes and edges, JNets can filter this network to create more focussed visualizations, for example, the networks in figures 5-7.

**Figure 5:**
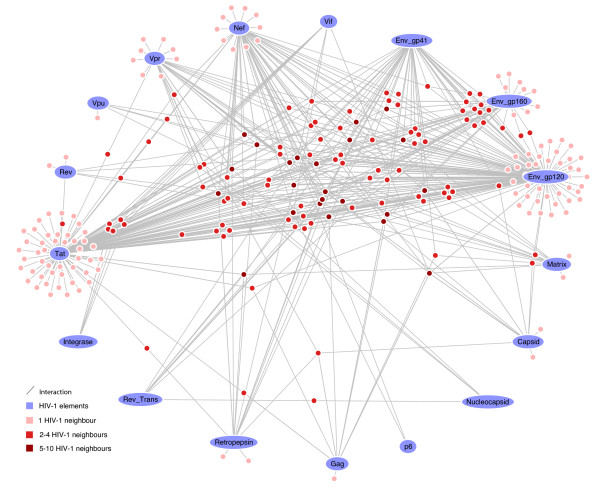
**HIV-1 interacting drug target genes**. This network shows 237 human genes that encode products that are HIV-1 interacting and FDA-approved drug targets. HIV-1 elements are labelled. Human genes are colored according to the number of distinct HIV-1 elements with which they share an interaction (darker = more). The layout of this network was achieved by manually positioning the HIV-1 nodes, locking them in position and allowing the JNets spring layout to reposition the remaining human gene nodes. As a result, human gene nodes with multiple interactors are drawn to the centre of the network. From this visualization it is clear that many HIDTs interact with multiple HIV-1 elements and that certain HIV-1 elements are responsible for many more HIV-HIDT interactions than others. Such observations can be investigated in greater detail using JNets. For example, 114 from 237 of these human genes interact with more than one HIV-1 element. This is significantly more than would be expected at random (*p *< 0.001).

The HIV-1-host, drug-target interaction network contained 3,492 nodes and 7,374 edges. Of the nodes, 19 were HIV-1 elements, 2,391 were human genes and 1,082 were drugs. Of the 2,391 human genes, 1,434 coded for HIV interacting products, 1,194 coded for approved drug targets and 237 coded for products that were both HIV-1 interacting and drug targets (throughout, we will refer to this gene set as HIDTs). HIDTs account for 17% of human genes that code for HIV-1 interacting products and 20% of the genes that code for drug targets. Of these 237 genes, we found gene-products of 178 to have one or more direct physical interactions with HIV-1, based upon the interaction type. This network also contained 7,374 edges: 3,939 HIV-host interactions and 3,435 drug-target interactions. The whole HIV-1-host, drug-target interaction network is displayed in figure 4, where HIDTs are highlighted. A second network view is shown in figure 5 in which JNets has been used to filter the network to show HIV-1 interactions with HIDTs. From this visualization, it is clear that many HIDTs interact with multiple HIV-1 elements and that certain HIV-1 elements (such as Env-gp120) are responsible for many more HIV-HIDT interactions than others (such as the p6 protein). These aspects of the network were investigated in more detail using JNets.

Of the 237 HIDTs, the gene-products from 114 interact with more than one distinct HIV-1 element. Using JNets, we showed that this is significantly greater than expected (Fisher's exact test, *p *< 0.001), given that from 1,434 human genes whose products are HIV-1 interacting, 529 have interactions with more than one distinct HIV-1 element. From 237 HIDTs, the gene products of 125 are targeted by more than one drug. By performing a Fisher's exact test on the drug-target network, we showed that this is a significantly greater proportion than expected at random (*p *< 0.001), given that 1,194 approved drug-target genes were identified in DrugBank and only 501 of these are targeted by more than one drug.

From 3,939 HIV-host interactions, 820 are between HIV-1 and HIDTs. We examined the HIV-1 elements that are responsible for these 820 interactions. We found significantly more Env-gp120 and Env-gp41 interactions and significantly fewer Tat and Integrase and Rev interactions among drug target genes, than would be expected, given the 3,939 HIV-human interactions as a parent population. See Table 1 for more details.

**Table 1:** HIV-1 interactions with approved drug target genes, by HIV-1 element.

HIV-1 element	Total interactions	Interactions with drug target genes	Corrected *p*
Env-gp120	856 (22%)	239 (29%)	5.85 × 10^-6^
Integrase	102 (3%)	5 (1%)	7.15 × 10^-4^
Rev	109 (3%)	7 (1%)	1.36 × 10^-3^
Env-gp41	190 (5%)	58 (7%)	4.65 × 10^-3^
Tat	1394 (35%)	249 (30%)	2.50 × 10^-2^
Capsid	45 (1%)	16 (2%)	6.23 × 10^-2^
Vif	77 (2%)	8 (1%)	6.57 × 10^-2^
Nef	302 (8%)	78 (10%)	7.60 × 10^-2^
Pol	1 (< 1%)	0 (0%)	0.238
Vpr	275 (7%)	47 (6%)	0.173
Env-gp160	213 (5%)	49 (6%)	0.432
Env-gp41	190 (5%)	58 (7%)	1.07 × 10^-2^
Rev	109 (3%)	7 (1%)	4.50 × 10^-3^
Integrase	102 (3%)	5 (1%)	2.61 × 10^-3^
Matrix	95 (2%)	15 (2%)	0.399
Retropepsin	89 (2%)	15 (2%)	0.506
Vif	77 (2%)	8 (1%)	0.120
Gag	72 (2%)	15 (2%)	0.889
Reverse transcriptase	45 (1%)	8 (1%)	0.605
Vpu	27 (1%)	5 (1%)	0.667
Nucleocapsid	26 (1%)	4 (< 1%)	0.486
p6	18 (< 1%)	2 (< 1%)	0.386
p1	3 (< 1%)	0 (0%)	0.253

The proportion of interactions that HIV-1 has with the products of genes that also encode drug targets, compared to total interactions, grouped by HIV-1 element are shown. The statistical significance of the difference in expected and actual figures, indicated by *p*-values, were calculated in JNets using two-tailed Chi-square tests, corrected for multiple testing using the Benjamini and Hochberg method [15]. Using a *p *cutoff of 0.05, we found significantly more Env-gp120 and Env-gp41 interactions and significantly fewer Tat, Integrase and Rev interactions among drug target genes, than would be expected at random.

Next, we examined the drugs that target the products of HIV-1-Interacting-DTs, compared to other drug target genes in the human genome. We found that drugs from certain drug classes, according to level-2    ATC classifications denoted in DrugBank, are more likely to target    gene products of HIDTs than would be expected at random, given all human drug target genes in DrugBank as a parent population. In total, we identified six such classes that showed over-representation (*p *< 0.001, table 2). We investigated three particular over-represented groups of genes in greater detail: (i) HIDTs that code for gene-products targeted by immunosuppressive agents, this was the most overrepresented drug category (*p *= 1.87 × 10^-13^), (ii) HIDTs that code for gene products targeted by antineoplastic agents, the second most over-represented category (*p *= 7.87 × 10^-9^), and (iii) HIDTs that code for gene-products targeted by statins – a specific subset of the category 'lipid modifying agents'. To identify as many HIDTs as possible whose products are targeted by these three drug types, we widened our search to take into account level-2 ATC codes *and *drug classifications from DrugBank. Immunosuppressants were defined with drug classification 'immunosuppressive agents', or, with the corresponding ATC level-2 classification 'L04'. Antineoplastic agents were defined with a drug classification containing the word 'antineoplastic', or, with the corresponding ATC level-2 classification 'L01'. Statins were defined with a drug classification of 'HMG CoA reductase inhibitors' or 'Hydroxymethylglutaryl-CoA Reductase Inhibitors', or, one of the level-3 ATC classifiers: 'C10AA' (HMG CoA reductase inhibitors), 'C10BA' (HMG CoA reductase inhibitors in combination with other lipid modifying agents) or 'C10BX' (HMG CoA reductase inhibitors, other combinations). After this redefinition, all three chosen drug groups were still statistically over-represented among drugs targeting the products of HIDTs, compared to other human drug target genes (*p *< 0.001).

**Table 2:** HIV-1 interacting drug target genes by drug category.

Level-2 ATC description	Target genes	HIV-1 interacting	Corrected *p*
Immunosuppressive agents	36 (3%)	29 (12%)	1.87 × 10^-13^
Antineoplastic agents	100 (8%)	47 (20%)	7.87 × 10^-9^
Anti-inflammitory and antirheumatic products	27 (2%)	18 (8%)	3.03 × 10^-6^
Stomatological preparations	30 (3%)	19 (8%)	3.35 × 10^-6^
Lipid modifying agents	46 (4%)	23 (10%)	4.62 × 10^-5^
Antithrombotic agents	65 (5%)	29 (12%)	4.08 × 10^-5^

The proportion of drug target genes that encode HIV-1 interacting products, compared to the total number of drug target genes, grouped by the level-2 ATC categories of targeting drugs are shown. The statistical significance of the difference in expected and actual figures, indicated by *p*-values, were calculated in JNets, using two-tailed Fisher's Exact tests, corrected for multiple testing using the Benjamini and Hochberg method [15]. We have only shown those categories where *p *< 0.001; all showed over-representation.

We found 21 HIDTs whose gene products are a target for HMG-CoA reductase inhibitors. This is 66% of all statin target genes given in DrugBank. This group shows a relatively high degree in the HIV-1-host network; the average degree of a host node is 2.8 but for this group of nodes the average degree was 6.7. From these 21 genes, 13 code for products that interact with Nef (over-represented where *p *< 0.001), 15 with Env gp120 and five with the Matrix protein (both over-represented where *p *< 0.05). In addition, these 21 HIDTs have certain over-represented interaction types with HIV-1 (*p *< 0.05): HIV-1 is activated by three, induces the release of five and upregulates the gene-products of 12 HIDTs in this group. Among these 21 HIDTs were the HMG-CoA reductase gene, whose gene-product is modulated by Nef and the ras homolog, member A (RhoA), whose gene-product is upregulated by Vpr and activated by Tat.

We found 73 HIDTs whose gene-products are a target for 81 different antineoplastic agents. This is 43% of all antineoplastic agent target genes given in DrugBank. From these 73 genes, the notable HIV-1 elements that their gene-products interact with are Env-gp120 (41 genes) and Nef (23 genes), which are over-represented (*p *< 0.05). From this group, no particular HIV interaction type was found to be statistically over-represented, after correcting for multiple tests. However, the most abundant classes were 'interacts with' (25 genes), upregulates (24 genes), activates (21 genes), inhibits (19 genes), binds (19 genes) and downregulates (12 genes). The 81 antineopastic agents that target the gene-products of HIV-1-Interacting-DTs were from a wide range of ATC classifications. From those with level 2 ATC class 'L01', corresponding specifically to antineoplastic agents, the most abundant were drugs from the 'L01X' level 3 ATC class, corresponding to 'other antineoplastic agents'. Therefore, a specific group of antineoplastic agents that target the same gene-products as HIV-1, was not easily identifiable.

**Figure 6:**
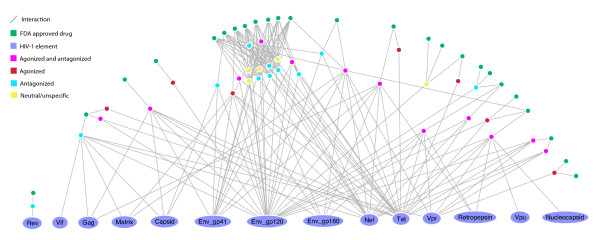
**Drug-target network showing all immunosuppressant target HIV-1 interacting genes**. The drug nodes lie in an arc around the top of the network. Between these two groups are the human gene nodes, colored according to the action HIV-1 has upon them. Four types of action are defined: agonised [7], antagonised [11], agonised and antagonised [12] and neutral/unspecified [5]. These distinctions are derived from the interaction description supplied with each HHPID HIV-1-host interaction. HIV-1 elements are shown at the bottom and are labelled. The human genes shown in this network are likely to perform a significant role in the immune system to be targeted by immunosuppressive drugs. From this visualization it is clear that HIV-1 targets many of the same proteins as immunosuppressant drugs.

**Figure 7:**
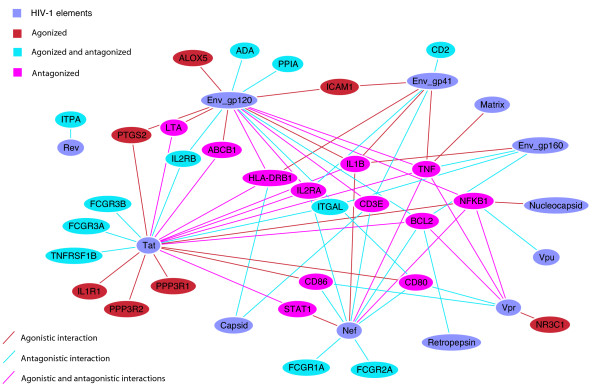
**HIV-1 host network showing immunosuppressive agent target genes**. Human genes that are both HIV-1 interacting and targeted by immunosuppressive agents are shown. Only those genes that are explicitly agonised or antagonised by HIV-1 have been included; human genes are colored according to this action. Seven host genes are agonised, eleven are antagonised and twelve are both agonised and antagonised. These distinctions are derived from the interaction description supplied with each HHPID HIV-1-host interaction.

Given the nature of HIV-1 infection, the immune cells that become infected and the detrimental effect that this infection can have on the immune response of the host, we expect to see a high proportion of immune system proteins among HIV-1 interacting drug targets. Indeed we found 35 genes in HHPID whose products are a target for immunosuppressants. This is 63% of all immunosuppressant target genes given in DrugBank. From these 35, the outstanding HIV-1 elements that they interact with, as for antineoplastic agent target genes, are Env-gp120 (25 genes) and Nef (15 genes), which are over-represented (*p *< 0.001). However, the products of these genes also interact with Env-gp41, 10 genes, and the capsid protein, four genes (*p *< 0.05). From this group of 35 genes, HIV-1 acts to downregulate the products of 16 (over-represented, *p *< 0.001). This was the only statistically significant HIV-1 interaction type detectable among this group of genes after correcting for multiple tests. However, HIV-1 also inhibits 12, upregulates 15 and activates the products of five genes from this group. Figure 6 shows the drug-target network, filtered to show only HIV-1-interacting immunosuppressant target genes, the immunosuppressive drugs and the HIV-1 elements that target the products of these genes. The human genes are colored according to the general action that HIV-1 infection has upon their products. Three categories are defined: agonised (e.g., activate, upregulate and enhance), antagonised (e.g., inhibit, downregulate and degrade), both agonised and antagonised, or neutral/unspecified (e.g., binds, modulates and interacts with). The agonised group contained 7 genes, the antagonised 11 genes, the agonised and antagonised 12 genes and five neutral genes, respectively. We found 25 immunosuppressive drugs that target gene products that are HIV-1 interacting, 18 of these drugs have more than one such target. The majority (14) of these 25 drugs have a level 4 ATC classification 'L04AA', meaning that they are selective immunosuppressants. Figure 7 shows the same results as in figure 6, however, for clarity, the drug nodes and human genes from the neutral group have been removed.

#### Case study: Discussion

As an example of the use of JNets, we have taken data from HHPID and DrugBank to research the cross section of genes that encode gene products that are both HIV-1 interacting and drug targets. We have shown that a significant proportion (237 genes, 17%) of genes that encode an HIV-1-interacting product also encode an approved drug target ('HIDTs'), of which 178 code for products that have direct physical interactions with HIV-1. In addition, a significant proportion of these interact with more than one HIV-1 element. This suggests that products of these genes are involved in important interactions with HIV-1, rather than being incidental effects of HIV-1 infection. Thus, drugging HIV-1 interacting proteins could potentially disrupt the HIV-1 life cycle. These host proteins could be explored in the search for new HIV-1 treatments.

Many of the HIV interactions involving HIDT gene products are with proteins from the Env complex, Nef and Tat. Tat, the *trans*-activator of transcription, promotes viral transcription and elongation [23]. As a consequence, Tat induces operation of the host transcription machinery and indirectly, the production of other viral proteins. Therefore, as a result of some direct interactions, Tat is also responsible for indirect, downstream responses in the host cell, many of which will be more directly due to the activity of transcription and the presence of other viral proteins. Moreover, Tat interactions were actually underrepresented among HIDTs. Therefore, it may not be Tat interacting human proteins that are most interesting from a drug-discovery perspective. In contrast, interactions with the Env complex were found to be over-represented among HIDTs. Env proteins and Nef are known to interact with host proteins located in the membrane. For example, the Envelope complex binds CD4 receptors and several co-receptors including CCR5 [24]. Nef, or negative factor, is known to downregulate CD4 [25] and class I MHC molecules [26]. Around 70% of drugs are believed to target membrane proteins [27], which may explain the prominance of Nef and Env interacting drug targets and the over-representation of Env interacting drug targets in our network.

Using JNets, we have shown that human genes that code for products targeted by HMG-CoA reductase inhibitors, antineoplastic agents and immunosupressive agents are over-represented in the HIV interaction data. HIV-1 interacts with the majority of targets of HMG-CoA reductase inhibitors, including HMG-CoA reductase itself. The HIV-1 virion requires clustering of host lipid 'rafts', also known as DIGs (detergent-insoluble glyco-lipid-enriched microdomains), for entry to and budding from the host cell [17]. Raft formation is believed to be controlled by Rho GTPases and remodelling of the actin cytoskeleton. HMG-CoA reductase is required to prenylate and activate these GTPases. Nef is thought to associate with these rafts and prime T-cells for activation and may promote HIV-1 replication [28]. However, due to the extensive crossover we have observed between the targets of HMG-CoA reductase inhibitor and HIV-1 interacting proteins, it seems likely that other mechanisms are active that give HMG-CoA reductase inhibitors the ability to decrease viral load in infected patients. One indicator of this is that the HMG-CoA inhibitor target genes have a particularly high degree in the HIV-1-host protein interaction network, suggesting that they are particularly important in the HIV-1 life cycle. Env-gp120 has interactions with the products of 15 of these genes, which is unsurprising when we consider that Env associates with many host membrane proteins and that these 15 host genes are likely to be involved in membrane raft formation and HIV-1 entry and exit from the cell. One notable interaction that Env has with a host protein is with coagulation factor II (thrombin). Thrombin has been shown to activate the Env complex and enhance fusion of the virus to the host cell [29]. Thrombin is also a target to the HMG-CoA reductase inhibitor simvastatin – a possible mechanism through which statins may be effective drugs in the treatment of HIV-1 infection.

From our results it is not clear why antineoplastic agents and HIV-1 share many human targets. By manually examining the group of drugs that target the products of these genes, it was noted that many are monoclonal antibodies, such as Trastuzumab (commonly known as Herceptin), that bind cell surface receptors. Trastuzumab action is believed to be involved with the mitogen-activated protein kinase (MAPK) cascade [30], a pathway with which HIV-1 infection is also highly associated, for example, the MAPK-1 gene has 22 distinct interactions with HIV-1 in our network, with 10 distinct HIV-1 elements. The systemic effects of antineoplastic agents and of HIV-1 infection may utilise many of the same intrinsic cellular pathways. AIDs-related malignancies are not an uncommon cause of death for HIV-1 infected patients [31,32]. This suggests that research in to the use of antineoplastic therapies in HIV-1 infected patients, may benefit from greater utilization of network-based approaches. Such approaches may lead to improved treatment strategies that avoid provoking greater levels of infection and virus-induced host cell perturbation. For example, to better understand crossover in specific cellular pathways, host proteins could be annotated with pathway data, points of contact with viral proteins, and drugs could then be identified and the network analysed further using JNets.

Our analysis has shown that the majority of the targets of immunosuppressive drugs are also HIV-1 interacting, an observation that we have shown to be statistically significant. In the case of HIV-1 infection, interaction with elements from the host immune response is particularly likely, as HIV infects cells that are specific to this host system, such as T-cells and macrophages. Therefore, HIV-1 requires a sufficient host immune response so that it has cells to infect but may also downregulate elements of the host immune response at the same time, presumably in order to evade other aspects of the immune system. This complexity is exemplified in our results, particularly in the network visualizations shown in figures 6 and 7. Figure 7 shows that HIV-1 elements, particularly Nef, Tat and Env, act to both agonise and antagonise proteins that are intrinsic to the host immune response. For example, there is no clear relationship that defines HIV-1 action on interleukin receptors (i.e., IL2RA, IL1RA, IL2RB). This could be due to a single, static network view being insufficient to expose variable aspects of HIV-1 infection, such as the point in the cell-cycle, the stage of HIV-1 infection and the infected cell type – it is inconceivable that these interactions all occur simultaneously in a single cell. Even so, this representation does suggest that HIV-1 possesses an intricate system for influencing the host immune response that is necessary to maintain the infection.

## Conclusion

In our case study, we have integrated data from DrugBank and HHPID to perform analyses. We have demonstrated that JNets is capable of combining biological annotation and interaction network data to allow specific visualizations to be created and statistical conclusions to be drawn, from which biological inferences can be made. However, JNets is not designed to compete with heavily developed network analysis packages, such as Cytoscape [8] and Pathway Studio [9]. These applications incorporate a plethora of functions for the analysis of biological networks. A drawback of such applications is that the utilities on offer may fall beyond the scope of many users, as adding greater functionality to software can complicate user interfaces and make simple tasks less accessible. Rather, JNets is designed to allow network visualization and some useful analysis to be carried out in a simple, web-deployable tool. By this system, vendors could use JNets as a 'gateway' to allow users to interact with, and better understand network data.

No part of JNets is specific to biological networks, making JNets useful across a wide range of disciplines. JNets is also configurable, to allow preset network views and subdivisions of networks to be stored. This aspect of the applet makes use of the JNets annotation filtering method, so that very different visualizations can be rendered from a single input file, available to the user at a mouse click. Much of the JNets system is based around the dynamic creation of subgroups by filtering network annotation. By offering these features, JNets is quite different from any other web deployable network visualization tool that is currently available. For example, WebInterViewer is designed to render a large number of nodes with great efficiency, although annotation can accompany the network [11]. VisAnt [10] analysis focusses on graph-theoretical properties and integration of annotation from predetermined sources. JSquid [13] was primarily designed as an interactive viewer for the FunCoup website , though it can be used for viewing independent network data. JSquid also makes use of node and edge groupings, however, unlike JNets, will not perform analyses on user defined features of these groups.

In summary, JNets is a platform independent, interactive network visualization and analysis tool, suitable for a wide range of network data and capable of being deployed from a website as a customized applet or run as a stand-alone application. For further information about JNets please visit .

## Availability and requirements

• **Project name**: JNets

• **Project home page**: 

• **Operating system(s)**: Platform independent

• **Programming language**: Java

• **Other requirements**: Java 1.5 or higher

• **License**: GNU GPL

• **Any restrictions to use by non-academics**: No

## Authors' contributions

JIM wrote the JNets software, carried out the analysis presented in the case study and wrote the manuscript. JWP provided help in producing the software. DLR conceived and supervised this study with the help of JWP. All authors read and approved the final manuscript.
